# Coral calcium carried hydrogen ameliorates the severity of non-alcoholic steatohepatitis induced by a choline deficient high carbohydrate fat-free diet in elderly rats

**DOI:** 10.1038/s41598-023-38856-6

**Published:** 2023-07-19

**Authors:** Kuai Ma, Xin Hu, Keiki Nambu, Daisuke Ueda, Naotsugu Ichimaru, Masayuki Fujino, Xiao-Kang Li

**Affiliations:** 1 Division of Transplantation Immunology, National Research Institute for Child Health and Development, 2-10-1 Okura, Setagaya-ku, Tokyo, 157-8535 Japan; 2 Acche Corporation, Tokyo, Japan; 3 Division of Hepato-Pancreato-Biliary Surgery and Transplantation, Department of Surgery, Kyoto University Graduate School of Medicine, Kyoto, Japan; 4 Department of Urology, Kinki Central Hospital, Hyogo, Japan; 5 Management Department of Biosafety, Laboratory Animal, and Pathogen Bank, National Institute of Infectious Diseases, 1-23-1, Toyama, Shinjuku-ku, Tokyo, 162-8640 Japan

**Keywords:** Non-alcoholic fatty liver disease, Non-alcoholic steatohepatitis

## Abstract

Hydrogen has been reported to act as an antioxidant, anti-apoptosis and anti-inflammatory agent. Coral calcium carried hydrogen (G2-SUISO) is a safer and more convenient form of hydrogen agent than others. The mechanism underlying the hepatoprotective effects of G2-SUISO using an elderly non-alcoholic steatohepatitis (NASH) rat model was investigated. Two days after fasting, six-month-old elderly male F344/NSlc rats were given a choline deficient high carbohydrate fat-free (CDHCFF) diet from day 0 to day 3 as CDHCFF control group, and then switched to a normal diet from days 4 to 7 with or without 300 mg/kg G2-SUISO. Rats in each group were finally being sacrificed on day 3 or day 7. In the CDHCFF diet group, G2-SUISO decreased the liver weight-to-body weight ratio, the serum AST, ALT, total cholesterol levels, inflammatory infiltration, pro-inflammatory cytokine expression and lipid droplets with inhibiting lipogenic pathways by reducing sterol regulatory element-binding protein-1c, acetyl-CoA carboxylase and fatty acid synthase gene expression compared with the CDHCFF diet alone. G2-SUISO had beneficial effects of anti-apoptosis as well the down-regulation of pro-apoptotic molecules including NF-κB, caspase-3, caspase-9 and Bax. These findings suggest that G2-SUISO treatment exerts a significant hepatoprotective effect against steatosis, inflammation and apoptosis in elderly NASH rats.

## Introduction

Non-alcoholic fatty liver disease (NAFLD) is the most common chronic liver disorder associated with metabolic dysfunction and is a leading cause of cirrhosis and hepatocellular carcinoma (HCC) with a global prevalence of 25%^1^. It usually develops in the absence of excessive alcohol consumption and is associated with an unhealthy diet and lack of physical activity. Non-alcoholic steatohepatitis (NASH) is a progressive form of NAFLD, characterized by chronic inflammation and hepatocyte injury due to fat accumulation^2^. A community-based study found that fatty liver is prevalent in the elderly population, with a prevalence of over 40%^3^.

Aging is a complex phenomenon characterized by the gradual decline of the tissue and organ function accompanying the irreversible age-related loss of viability. Impairment of the liver function and development of NAFLD are common among the elderly^4^. Accumulating evidence has pointed out that the process of aging itself markedly increases the prevalence of metabolic syndrome in humans, reportedly being a risk factor of NAFLD^5^, as it predisposes individuals to hepatic functional and structural impairment and metabolic risk. Oxidative stress is considered the primary cause of general aging as well as diseases associated with aging, especially metabolic diseases^6^. Oxidative stress, lipotoxicity and inflammation^7^ have been shown to play central roles in the development and progression of NAFLD. Furthermore, the progressively increased production of reactive oxygen species (ROS) during the aging process contributes to the accumulation of lipids, particularly cholesterol, in the liver of elderly individuals^8^.

Molecular hydrogen (H_2_) was previously reported to act as an antioxidant for preventive and therapeutic applications by selectively alleviating cytotoxic oxygen radicals without affecting other ROS^9^. Previous studies have reported that H_2_ functions as an antioxidant, anti-apoptosis and anti-inflammatory agent in many animal models and human clinical studies^10^. Among these studies, H_2_ administration can be roughly divided into inhaling H_2_ gas, drinking H_2_ dissolved water and injecting H_2_ dissolved saline^11^. In our present study, we used coral calcium carried hydrogen (G2-SUISO), which is a safer and more convenient form of H_2_ agent than others, as the source of H_2_. In general, H_2_ cannot be kept in supplements as-is. Coral powder was therefore selected as the material to convey H_2_, and we employed a unique method to successfully immobilize H_2_ on the surface of the carrier coral calcium. When the coral calcium enters the body, hydrogen is generated upon contact with moisture.

The present study investigated the potential effects of G2-SUISO with the goal of evaluating whether or not G2-SUISO could attenuate the severity of NASH induced by a choline deficient high carbohydrate fat-free (CDHCFF) diet in elderly rats.

## Results

### G2-SUISO attenuated CDHCFF-induced nonalcoholic steatohepatitis

**Figure 1:**
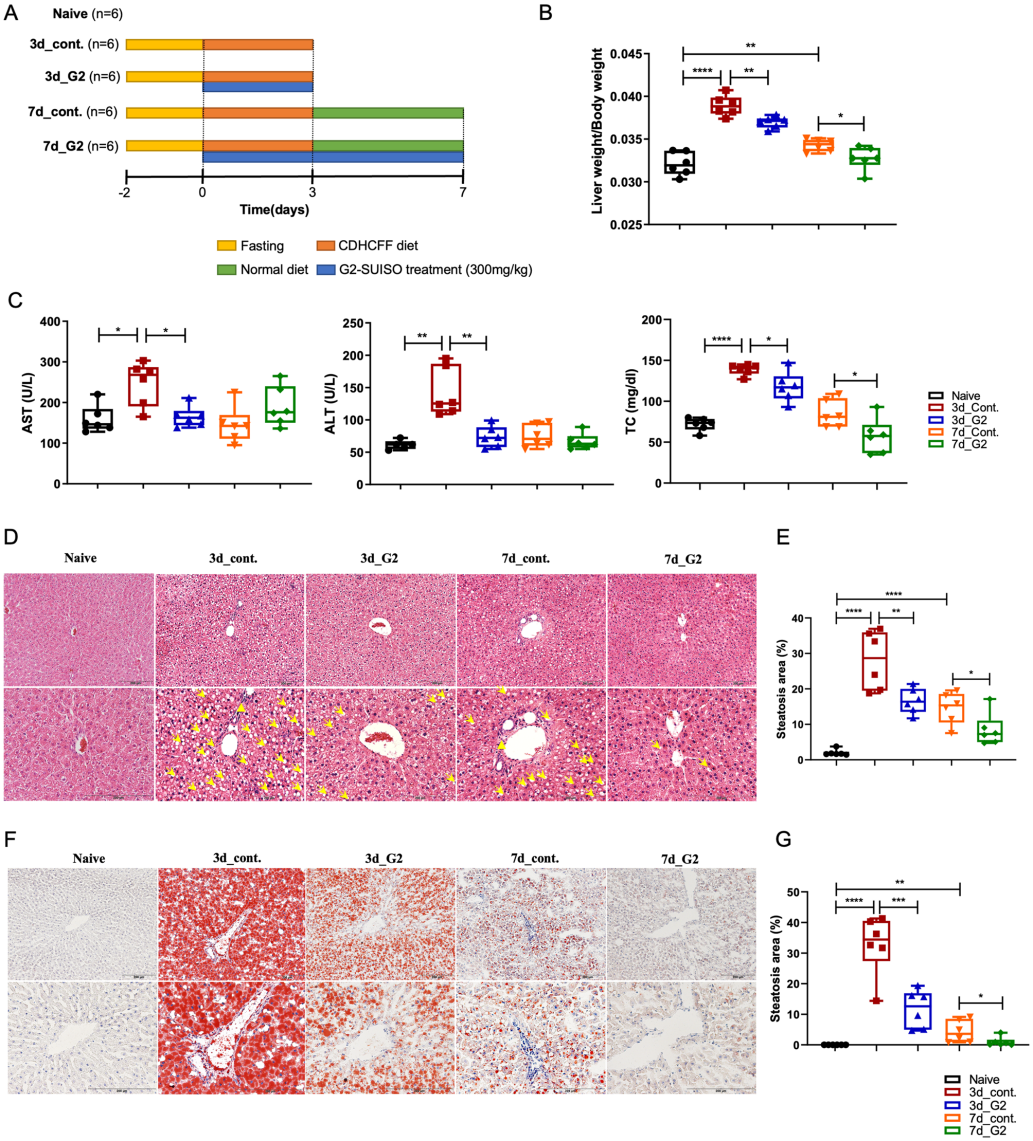
G2-SUISO attenuated CDHCFF-induced nonalcoholic steatohepatitis. (**A**) The experimental design and timeline of the groups was shown. (**B**) The liver weight-to-body weight ratio in the five groups is shown. (**C**) G2-SUISO treatment significantly reduced serum ALT, AST and TC levels in NASH rats. (**D**) Hematoxylin and eosin (HE) staining of liver specimens in different groups suggested that G2-SUISO improved hepatic steatosis in NAFLD rats. The yellow triangle represents the area of inflammatory cell infiltration. White vacuoles showed lipids (yellow arrow) in HE staining (magnification × 100 & × 200). (**E**) An analysis of the HE staining of fatty liver specimens is shown. Each bar represents the mean ± SD. (**F**) Liver sections of five groups were stained by Oil Red O solution. Red areas showed lipids in Oil Red O staining (magnification × 100 & × 200). (**G**) An analysis of the Oil Red O staining of fatty liver specimens is shown. Each bar represents the mean ± SD; *p < 0.05, **p < 0.01, ***p < 0.001, ****p < 0.0001.

As shown in Fig. 1B and Supplementary Fig. 3A, the liver weight-to-body weight ratio in the 3d_control group was significantly higher (*p* < 0.0001) than that in the Naïve group, while this value was lower (*p* < 0.01) in the 3d_G2 group. Furthermore, the liver weight-to-body weight ratio in the 7d_G2 group was down-regulated (*p* < 0.05) compared with the 7d_control group as well. The above results suggested that CDHCFF administration resulted in liver enlargement, and going back to eating normal diet again with G2-SUISO treatment significantly attenuated this CDHCFF-induced liver enlargement.

NASH is characterized as the excessive accumulation of TC in lipid droplets (LDs) in hepatocytes, and ALT and AST activities are important biomarkers of liver damage or diseases^8^. G2-SUISO significantly reduced the serum ALT, AST and TC levels in elderly NASH rats (Fig. 1C). The serum ALT and AST levels both notably differed between the 3d_control group and 3d_G2 group (*p* < 0.001), indicating that a CDHCFF diet caused severe hepatocellular injury in rats. Compared with the 3d_control group, the serum ALT and AST levels in the 3d_G2 group were markedly decreased. In addition, the serum TC levels were higher in the 3d/7d_control group than in the 3d/7d_G2 group (*p* < 0.05 and *p* < 0.05, respectively) suggesting that G2-SUISO had notable effects of attenuating CDHCFF-induced NASH. In contrast, serum triglyceride (TG) was significantly decreased by CDHCFF diet and G2-SUISO treatment showed comparable concentrations (Supplementary Fig. 3B). Based on the HE staining of liver specimens in Fig. 1D, we observed that the normal liver showed a clear and homogeneous texture, while the CDHCFF groups developed hepatocyte steatosis, ballooning and inflammatory cell infiltration on day 3 that was relieved on day 7. Compared with the CDHCFF diet groups, hepatocyte ballooning and steatosis in rat specimens were clearly reduced in the G2-SUISO groups on days 3 and 7 (Fig. 1E, *p* < 0.001, *p* < 0.001, respectively), suggesting that G2-SUISO ameliorated hepatic steatosis in NASH rats.

To further explore the protective effects of G2-SUISO on reducing steatosis in elderly NASH rats, liver sections from the five groups were subjected to Oil red O staining, which was used to measure fat loading in the hepatocytes. Based on Fig. 1F, we can see that G2-SUISO decreased intracellular lipid deposition in the livers of the CDHCFF group. A histological analysis of Oil Red O staining revealed a significant increase in intracellular lipid deposition in the livers of the 3d_CDHCFF group and 7d_CDHCFF group (*p* < 0.0001 and *p* < 0.01, respectively) (Fig. 1G). Numbers of LDs were markedly reduced in the livers of G2-SUISO-treated mice on days 3 and 7 (*p* < 0.001 and *p* < 0.05, respectively).

### G2-SUISO exerted protective effects against inflammation

**Figure 2:**
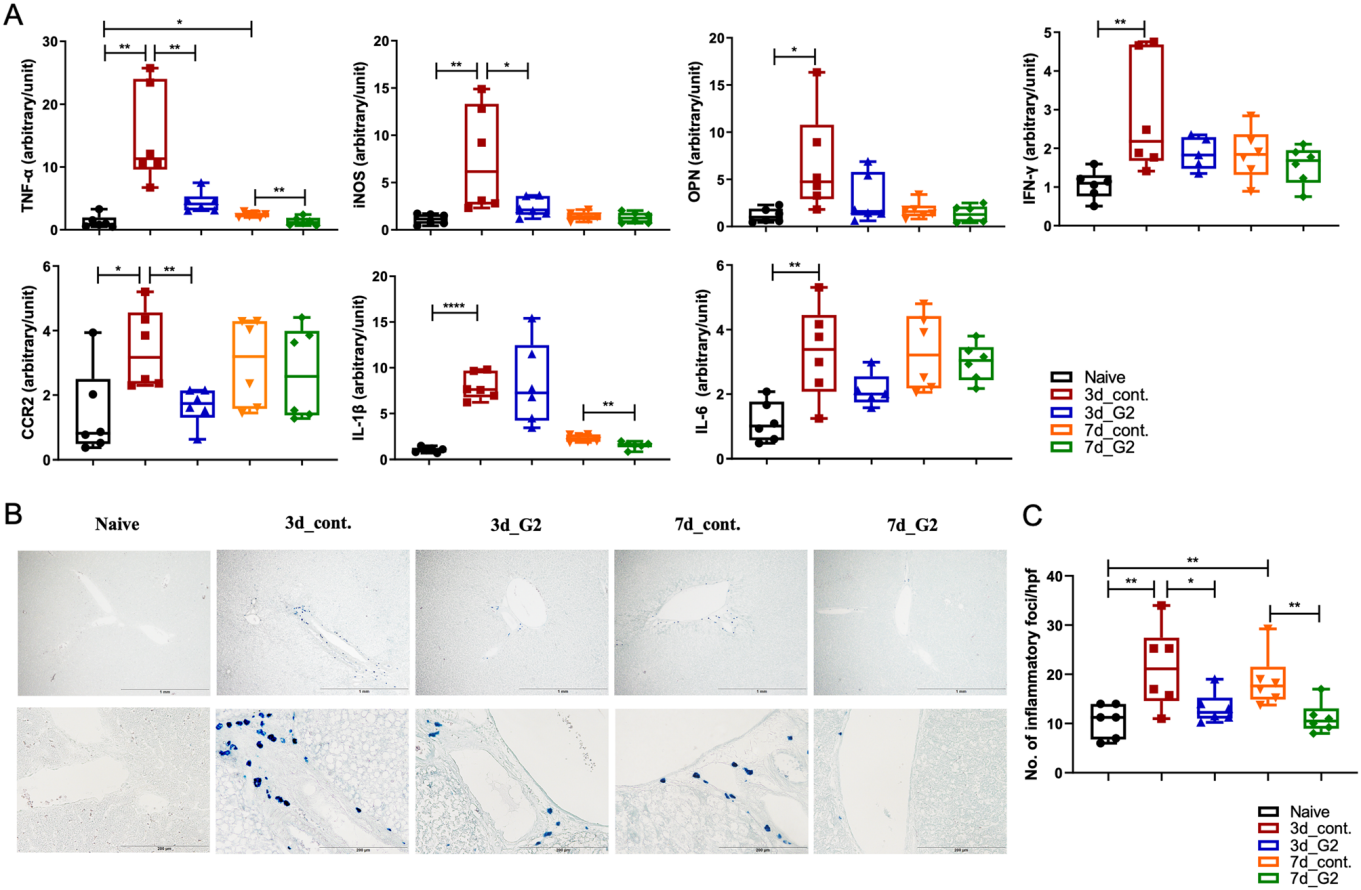
G2-SUISO reduced the mRNA expression of inflammatory cytokine-related genes. (**A**) Homogenates of liver tissues were analyzed by qRT-PCR, as described in the Materials and Methods. The mRNA expression of inflammatory cytokine-related genes, particularly TNF-α, iNOS and CCR2, was significantly lower in the 3d_G2 group than in the 3d_control group. The mRNA expression of inflammatory cytokine-related genes, such as IFN-γ, OPN, IL-1β and IL-6, tended to be down-regulated following G2-SUISO treatment. Values are expressed as the mean ± SD in arbitrary units; *p < 0.05, **p < 0.01, ****p < 0.0001. (**B**) Chloroacetate esterase staining of liver specimens with inflammatory foci in the five groups is shown (magnification × 40 & × 200). (**C**) Analysis results of chloroacetate esterase staining of fatty liver specimens are shown. A total of 4 high power fields (hpf) (× 40) were randomly selected from each liver specimens (n = 5), and the number of inflammatory foci was counted. The data are expressed as the cell number/high-power field. Each bar represents the mean ± SD; *p < 0.05.

Among cytokine-related to the progression of NASH, tumor necrosis factor-alpha (TNF-α) plays a pivotal role in the inflammatory pathogenesis of NASH^12^. As shown in Fig. 2A, hepatic mRNA expression of inflammatory cytokine-related genes, particularly TNF-α, inducible nitric oxide synthase (iNOS), osteopontin (OPN), interferon-gamma (IFN-γ), interleukin-1 beta (IL)-1β, IL-6 and C–C chemokine receptor type 2 (CCR2), were significantly higher following administration of an CDHCFF diet. In contrast, the expression of TNF-α, iNOS and CCR2 was lower in the 3d_G2 group than in the 3d_control group (*p* < 0.01, *p* < 0.05 and *p* < 0.01, respectively). The IFN-γ, OPN, IL-1β and IL-6 expression was down-regulated as well but without a significant difference. After the administration of G2-SUISO for 7 days, TNF-α and IL-1β showed notable reductions in expression (*p* < 0.01 and *p* < 0.01, respectively). The infiltration of neutrophils in liver was assessed using chloroacetate esterase staining of liver specimens. The hepatic expression of neutrophils was significantly increased in the CDHCFF diet control group compared with Naïve group on days 3 and 7 (*p* < 0.05 and *p* < 0.05, respectively) (Fig. 2B,C). Following the administration of G2-SUISO, the neutrophil numbers in NASH liver specimens were reduced on days 3 and 7 (*p* < 0.05 and *p* < 0.05, respectively), and only a few scattered inflammatory foci were observed compared with the control group. In addition, the infiltration of T cells and macrophages in the liver was also analyzed using CD3 and ED1 monoclonal antibody. Both expressions in the liver specimens were increased in the 3d_CDHCFF group compared to the Naïve group (*p* < 0.01 and *p* < 0.01, respectively), and G2-SUISO treatment decreased the infiltration of CD3- and ED1-positive cells (Supplementary Fig. 2). Taken together, these findings suggested that G2-SUISO might prevent inflammation and inflammatory cell infiltration in the NASH elderly model liver.

### G2-SUISO exerted anti-apoptotic effects

**Figure 3:**
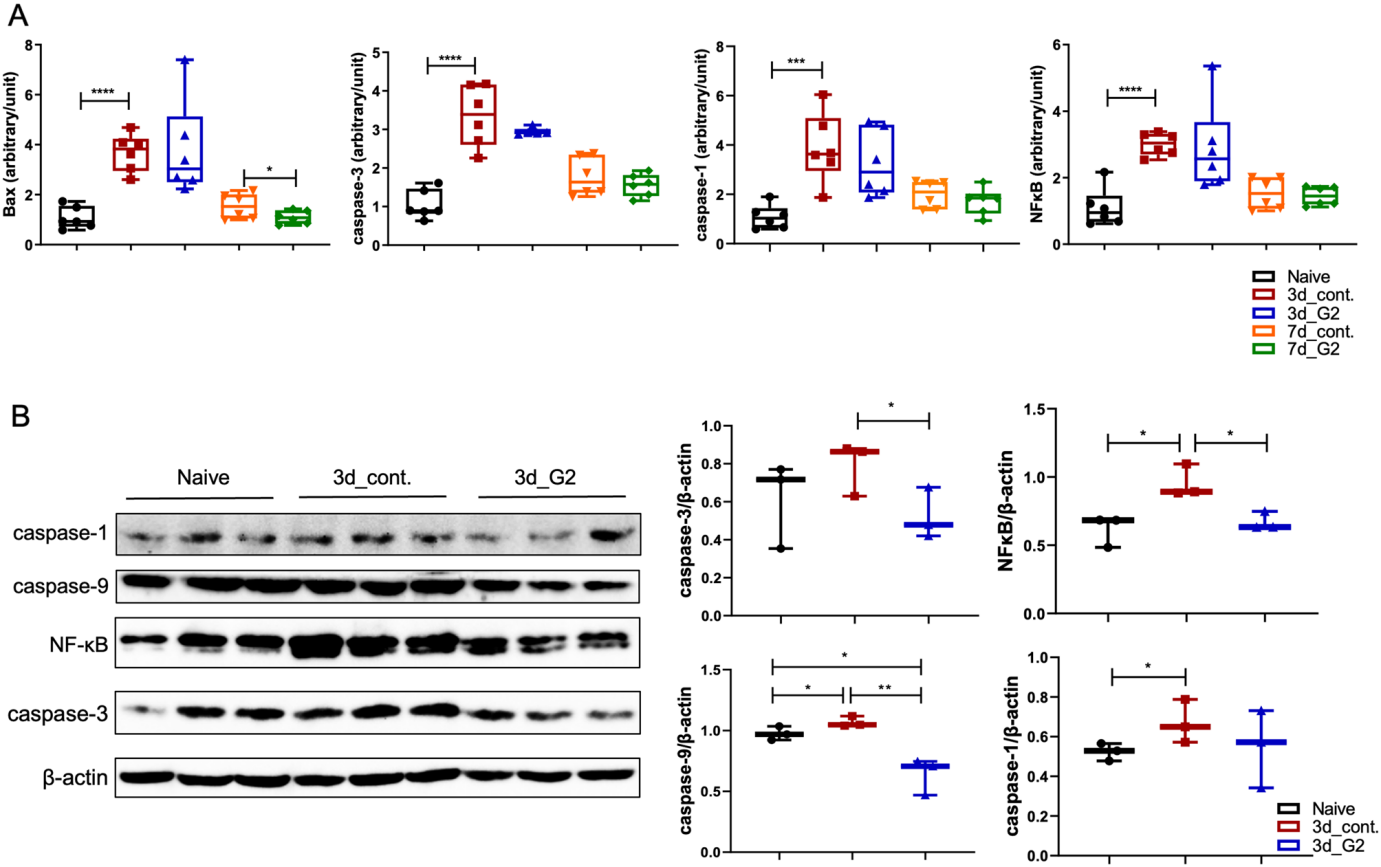
G2-SUISO reduced the mRNA expression of apoptotic molecules. (**A**) The mRNA expression of apoptosis-related genes in the five groups, particularly Bax, caspase-1, caspase-3 and NF-κB, is shown. (**B**) Results of a Western blot analysis of caspase-1, caspase-3, caspase-9 and NF-κB levels in the liver tissue of the Naïve group, 3d_control group and 3d_G2 group are shown. Data are expressed as the mean ± SD; *p < 0.05, **p < 0.01, ***p < 0.001, ****p < 0.0001.

Previous studies have reported that increased hepatocyte apoptosis may play an important role in controlling the development of NASH^13^. As shown in Fig. 3A, the mRNA expression of apoptosis-related molecules, particularly Bax, caspase-1, caspase-3 and NF-κB, was markedly up-regulated in the 3d_CDHCFF control group compared with Naïve group (*p* < 0.0001, *p* < 0.001, *p* < 0.0001 and *p* < 0.0001, respectively). The mRNA expression of Bax showed a significant decrease (*p* < 0.05) in the 7d_G2 group, and that of caspase-1, caspase-3 and NF-κB showed decreasing trend. Furthermore, the mRNA expression of caspase-3 in the 3d_G2 group showed decreasing trend compared with the 3d_control group.

To confirm the anti-apoptosis effect of G2-SUISO, we measured the protein levels of caspase-1, caspase-3, caspase-9 and NF-κB in liver tissues in the Naïve group, 3d_control group and 3d_G2 group by Western blotting (Fig. 3B). After a densitometric analysis of the signals, we found that the expression of caspase-3, caspase-9 and NF-κB was significantly reduced by the treatment of G2-SUISO (*p* < 0.05, *p* < 0.01 and *p* < 0.05, respectively), whereas the caspase-1 expression showed no significant difference from before treatment.

### G2-SUISO reduced steatosis in CDHCFF-induced NASH

**Figure 4:**
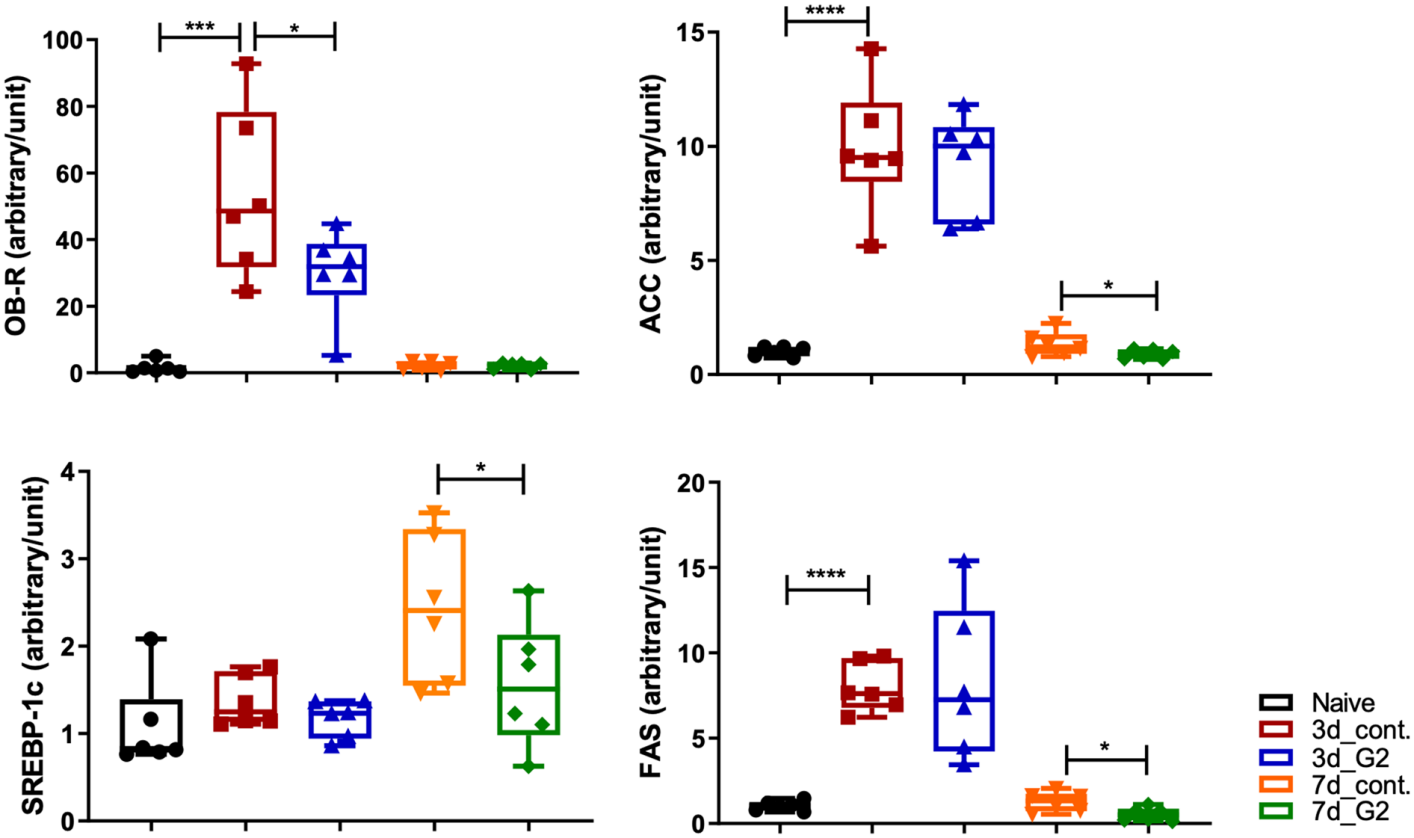
G2-SUISO reduced hepatocyte steatosis in CDHCFF diet-induced nonalcoholic steatohepatitis rat. The mRNA expression of fatty acid uptake- and lipid metabolism-related genes in the five groups, particularly leptin receptor (OB-R), fatty acid synthase (FAS) and acetylCoA carboxylase (ACC), as well as sterol regulatory element-binding protein-1c (SREBP-1c) are shown. Data are expressed as the mean ± SD; *p < 0.05, **p < 0.01, ***p < 0.001, ****p < 0.0001.

As a pathological analysis showed that G2-SUISO reduced the lipid deposition caused by an CDHCFF diet in the liver (Fig. 1C–F), the mRNA expression of fatty acid uptake- and lipid metabolism-related cytokine-related genes in the five groups, particularly leptin receptor (OB-R), fatty acid synthase (FAS) and acetylCoA carboxylase (ACC), as well as sterol regulatory element-binding protein-1c (SREBP-1c) was measured. In Fig. 4, the mRNA expression of the OB-R, ACC and FAS genes increased significantly in the 3d_CDHCFF control group compared with the Naïve group (*p* < 0.001, *p* < 0.0001 and *p* < 0.0001, respectively). The SREBP-1c gene expression in the 3d_CDHCFF control group also showed an increasing trend but without significance. G2-SUISO markedly down-regulated the expression of OB-R compared with the 3d_CDHCFF control group (*p* < 0.05) and tended to down-regulate the expression of ACC and SERBP-1c. After 7 days of G2-SUISO administration, the mRNA expression of SREBP-1c, ACC and FAS showed significant reductions (*p* < 0.05, *p* < 0.05 and *p* < 0.05, respectively). Furthermore, the mRNA expression of cholesterol metabolism genes, such as sterol regulatory element binding protein-2 (SREBP-2) or hydroxymethyl-glutaryl-CoA reductase (HMGCR) genes was increased by CDHCFF diet and G2-SUISO treatment showed the trend of decrease of the expression of these mRNA expression (Supplementary Fig. 3C).

## Discussion

The prevalence of NAFLD has increased significantly in parallel with increasing rates of obesity, now being the most common cause of chronic liver disease worldwide^14^. NAFLD is reported to be a heterogeneous disease with a high prevalence in elderly patients, characterized by the accumulation of TG and fatty acids in hepatocytes^15^. Compared with younger groups, NAFLD in the elderly may carry a more substantial burden of hepatic and extra-hepatic manifestations and complications^16^. Indeed, in our NASH model, aged rats showed more severe hepatitis when fed an CDHCFF diet than young rats (Supplementary Fig. 1). H_2_ has been reported to act as a therapeutic antioxidant by selectively reducing cytotoxic oxygen radicals, potentially leading to therapeutic effects in a variety of diseases, such as ischemia–reperfusion injury, colitis, NASH and aging-related diseases ^9,17–20^. G2-SUISO is widely used in dietary supplement products (https://acche.co.jp/supplement/items_platinum/) as a safe and effective form of antioxidant with minimal side effects. This study demonstrated the promising potential effects of G2-SUISO in a rat elderly model of NASH.

In this study, we used a CDHCFF diet, which is one of the useful methods, to generate our NASH rat model^21,22^. As expected, CDHCFF diet-fed rats exhibited increased adipose tissue weights and liver weight-to-body weight ratios compared to the Naïve group (Fig. 1). In addition, the liver pathology summarized the major features of human NASH, including steatosis, ballooning degeneration and inflammation. With this model, the hepatic lipogenic/inflammation/apoptosis gene expression and serum biochemical markers, such as AST, ALT and TC, attenuation by G2-SUISO showed convincing results for estimating the effect of the drug in our research. Oxidative stress and inflammation are the main components that contribute to the pathogenesis of NASH. It is widely acknowledged that TNF-α expression increases in cases of obesity and plays a major role in the inflammatory pathogenesis of NASH^23^. Enrichment of innate immune cells and increased inflammation are hallmarks of NASH. Increasing evidence supports that neutrophils play a key role in the onset of NASH, and histological findings from human liver biopsies suggest that enhanced infiltration of neutrophils is one of the key histological features of NASH^24,25^. Activation of the transcription factor NF-κB also results in production of key chemokines for neutrophil recruitment^26^. Steatosis is reported to lead to increased signaling of the transcription factor NF-κB, which can induce the production of pro-inflammatory mediators, such as TNF-α, IL-6 and IL-1β^27^. In addition, these pro-inflammatory cytokines contribute to the recruitment and activation of Kupffer cells to mediate inflammation in NASH. In our study, the mRNA expression of TNF-α significantly decreased following the administration of G2-SUISO to our rat NASH model (Fig. 2). The expression of other pro-inflammatory mediators, including IFN-γ, OPN, IL-1β, CCR2, iNOS and IL-6, was markedly increased under CDHCFF diet. Following G2-SUISO treatment, most of these cytokines showed a down-regulated trend, suggesting that G2-SUISO might have an anti-inflammatory effect. As shown in Fig. 3, down-regulation of NF-κB by G2-SUISO is one of the possible reason for the reduction of pro-inflammatory molecules, while the mechanism of NF-κB suppression by H_2_ is still unclear^28^. Other reasons may include induction of anti-inflammatory molecules. The trend toward enhanced mRNA expression of HO-1 was observed by G2-SUISO administration (data not shown) and several studies demonstrated that HO-1 inhibit NF-κB^29–31^.

Previous studies have reported that cell death, including apoptosis, seems to play a vital role in the progression of NASH^13^. Apoptotic hepatocytes stimulate immune cells and hepatic stellate cells (HSCs) to progress to NASH and fibrosis through the production of inflammasomes and cytokines. NF-κB is a master regulator of inflammation and cell death in the development of various liver diseases, such as NAFLD, hepatocellular injury, liver fibrosis and HCC^32^. The activation of NF-κB in Kupffer cells or infiltrating monocytes is pro-inflammatory and induces the expression of death ligands, such as TNF-α^33^. Caspases are related to the induction of apoptosis, which is a mode of cell death regulated by homeostasis, supporting the coordinated demolition and clearance of aging and damaged cells^34^. Bax belongs to the Bcl-2 protein family, and its pro-apoptotic function has been confirmed in many studies^35^. The expression of these pro-apoptotic molecules was significantly up-regulated in the CDHCFF diet control group on day 3 (Fig. 3). The administration of G2-SUISO then down-regulated the pro-apoptosis molecules, such as NF-κB and caspases, on day 3 according to our Western blot analyses. These findings suggest that G2-SUISO may prevent apoptosis in the NASH model liver by inhibiting the expression of pro-apoptotic molecules.

In the present study, G2-SUISO attenuated lipid accumulation in CDHCFF-induced NASH in elderly rats. The reverse alterations in the hepatic lipid accumulation can be explained by the effects of G2-SUISO on lipid metabolism. Previous studies have shown that the excessive hepatic accumulation of TG and FFAs induces hepatic steatosis^36,37^. The present study demonstrated that treatment with G2-SUISO ameliorated the lipid accumulation in the liver of CDHCFF diet rats via the modulation of lipid metabolism-related molecules. The hepatic uptake of fatty acids is thought to occur via several mechanisms, including a transporter-mediated mechanism. In patients with NAFLD, the hepatic expression of fatty acid synthesis genes and fatty acid oxidation-related genes is up-regulated. ACC catalyzes the production of malonyl-CoA and is a major building block for de novo lipogenesis, promoting the oxidation of FFAs^38^. SREBP-1c is a transcription factor that is a major regulator of FAS and other lipogenic proteins and is essential for the utilization and storage of glucose carbon^39^. It regulates the onset of the lipogenic program and is able to bind to the promoters of several lipogenesis enzyme genes and induce their expression^40^. The activity of the SREBP-1c/FAS pathway was previously shown to be markedly elevated and to contribute to the progression of hepatic steatosis in NASH mice^41^. Our present findings showed that G2-SUISO significantly down-regulated SREBP-1c, FAS and ACC expression (Fig. 4), indicating that G2-SUISO protects NASH rats from the SREBP-1c/FAS pathway. In this study, serum TG remained reduced after 2 days of fasting and feeding the CDHCFF diet, with comparable concentrations with/without G2-SUISO (Supplementary Fig. 3B). Previous study demonstrated that serum TG concentration was decreased after 2 days fasting but gradually increase after refeeding^21^. The reason why serum triglyceride levels are not increased by the CDHCFF diet is still unclear, but one possible reason may be due to the use of rats of different species and ages in this study.

**Figure 5:**
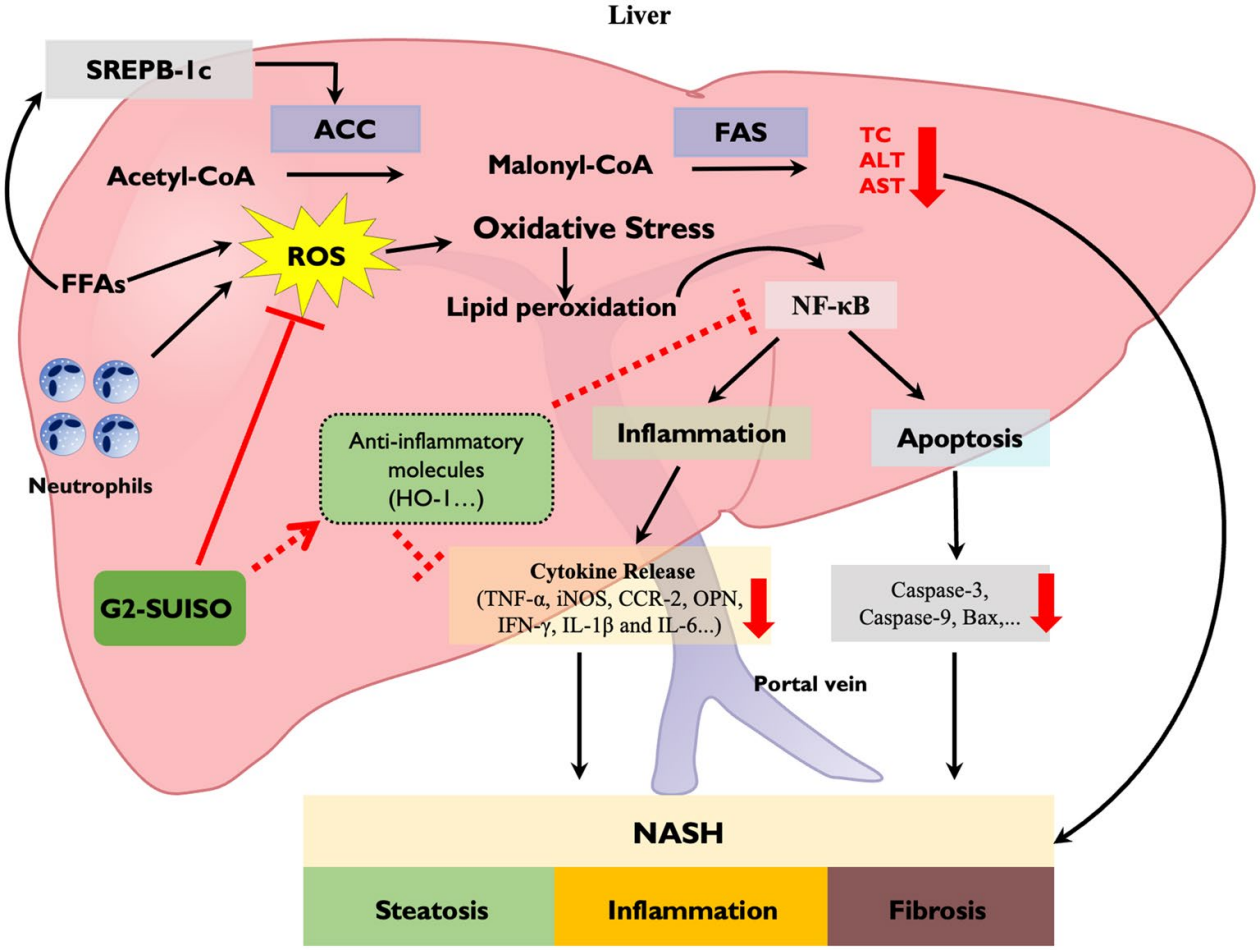
Schematic hypothesis of the mechanisms underlying the effects of G2-SUISO for treating nonalcoholic steatohepatitis. The beneficial effect of G2-SUISO against hepatic steatosis in NASH elderly rats may occur through the inhibition of lipogenesis pathways by reducing SREBP-1c, ACC and FAS gene expression, thereby causing a reduction in the hepatic fat accumulation and a significant decrease in total cholesterol (TC) levels in serum. The administration of G2-SUISO can decrease lipid peroxidation and pro-inflammatory cytokines, such as TNF-α, iNOS, CCR-2, IFN-γ, OPN, IL-1β and IL-6, which modulate liver damage in CDHCFF diet-fed rats. G2-SUISO might also up-regulated anti-inflammatory molecules, such as HO-1, which suppressed NF-κB and inflammatory cytokine expression. G2-SUISO is therefore able to reduce the activities of AST and ALT in the serum of NASH elderly rats. Furthermore, G2-SUISO was found to exert anti-apoptotic effects as well by down-regulating pro-apoptotic molecules, such as caspase-9, caspase-3 and Bax via down-regulation of NF-κB. Overall, this study provides evidence for the beneficial effects of G2-SUISO in reversing the progression of NASH in elderly rats.

As shown in Fig. 5, hepatic FFAs in the liver were increased after feeding an CDHCFF diet and accounted for the majority of the lipid accumulation, which can trigger NASH^42,43^. Excessive consumption and dietary abnormalities (such as consuming an CDHCFF diet after fasting) is related to oxidative stress in various tissues, including vessels, adipose tissues and the liver, and is consequent to disease development. Normally, oxidative stress, such as ROS, is continuously generated within cells but is counterbalanced by the antioxidant system to defend the body from cellular or tissue damage^44^. In the progression of aging and lipogenesis, an imbalance of oxidant synthesis and antioxidants is the major contributor to the pathogenesis of NASH, leading to liver injury and hepatocyte deterioration^45^.

Antioxidants have been suggested to be beneficial for health promotion and disease prevention. Chemiluminescence emission in vitro has been used to verify that H_2_ can scavenge ROS markedly^46^. Our results confirmed that the administration of G2-SUISO, a proven safe and convenient antioxidant, improved NASH in our elderly rat model, probably due to its antioxidant activity. Hepatic and general serum marker levels including the liver weight-to-body weight ratio, AST and ALT were all improved, while FFA uptake-related, inflammatory and pro-apoptosis molecules were suppressed in the NASH liver by administration of G2-SUISO. The beneficial effects of G2-SUISO against hepatic steatosis in NASH elderly rats may be exerted through the inhibition of lipogenesis pathways by reducing SREBP-1c, ACC and FAS expression, thereby causing a reduction in the hepatic fat accumulation and a significant decrease in TC levels in serum. Overall, these results indicate that G2-SUISO represents a simple and novel therapeutic strategy for NASH and NAFLD. Previous studies also showed that H_2_ therapy is a very promising treatment of liver diseases and the rational use of it has already solved many problems clinically^47^. However, the current clinical delivery method of H_2_ is not very convenient, and G2-SUISO can be made into capsules to solve this problem and facilitate H_2_ administration.

## Methods

### Manufacturing method of G2-SUISO

The original method of coral calcium carried hydrogen was described previously^48,49^. The coral powder containing calcium carbonate was sealed into a pressure vessel, and gas with a concentration of 100% (vol) H_2_ was circulated at a rate of 5 L/min at a temperature of 800 °C and pressure of 0.8 MPa, treated at a high temperature for 1 h. At 300 °C and 0.8 MPa, H_2_ gas concentration of 100% (vol) was circulated at a speed of 5 L/min and treated at a low temperature for 4 h. Finally, hydrogen powder with an average particle size of about 10 μm was obtained by grinding.

### Animal model

Six-month-old elderly male F344/NSlc rats (450–500 g) were purchased from Shizuoka Laboratory Animal Center (Shizuoka, Japan) and housed in a feeding room with automatically controlled light and temperature according to the guidelines of the Institutional Animal Care and Use Committee. All animal procedures were authorized by the National Research Institute for Child Health and Development (Permission No. A2020-004-C01-M01).

An acute NASH model was originally developed in 1997^21^ and is currently used in the field of fatty liver research with minor modification^22,50–52^. As early as three days after starting the CDHCFF diet, rats may develop hepatic inflammation. In our study, acute NASH in a rat model was induced by fasting for two days followed by feeding a CDHCFF diet for three days. Rats in the present study were randomized into five groups as shown in Fig. 1A and as follows: (1) Naïve group (n = 6): rats received a normal diet and were gavaged with distilled water; (2) CDHCFF control group on day 3 (3d_cont.) (n = 6): rats were fed an CDHCFF diet from days 0 to 3 and then sacrificed on day 3; (3) CDHCFF + G2-SUISO-treated group on day 3 (3d_G2) (n = 6): rats had NASH induced, were gavaged with 300 mg/kg G2-SUISO from days 0 to 3, and then were sacrificed on day 3; (4) CDHCFF control group on day 7 (7d_cont.) (n = 6): rats were fed an CDHCFF diet from days 0 to 3, switched to a normal diet from days 4 to 7, and then were sacrificed on day 7; (5) CDHCFF + G2-SUISO-treated group on day 7 (7d_G2) (n = 6): rats were fed an CDHCFF diet from days 0 to 3 and then switched to a normal diet from days 4 to 7. At the same time, rats were gavaged with 300 mg/kg G2-SUISO from days 0 to 7 before finally being sacrificed on day 7. At sacrifice, blood was collected, the liver weight-to-body weight ratio was measured, and the entire liver was removed for further analyses.

### Serum biochemical analyses

Serum was collected from whole-blood samples after standing for 30 min at 37 °C and centrifuged at 3000 g for 20 min at 4 °C. The samples were then measured for the AST, ALT, TC and TG concentrations with a commercially available kit (Fujifilm, Tokyo, Japan) and an automatic biochemical analyzer (DRI-CHEM 3500i; Fujifilm) according to the manufacturer’s instructions.

### Histology and histopathological analyses

Liver tissues were cut and fixed in 10% formaldehyde solution for 48 h and embedded in paraffin for histological analysis. Sections of liver 4-μm-thick were prepared and subjected to staining with hematoxylin and eosin (HE) (Muto Pure Chemicals, Tokyo, Japan) for morphological analyses to evaluate hepatocyte steatosis, ballooning and inflammatory cell infiltration. For another assessment of inflammatory cell infiltration, the quantification of neutrophils in liver specimens was stained using the Naphthol AS-D Chloroacetate Esterase Staining Kit (Muto Pure Chemicals). Slides were then examined by light microscopy (OLYMPUS, Tokyo, Japan) in a blind fashion to assess the inflammation state. Histological results were quantified using the WinRoof 7.4 software program (Mitani Corporation, Tokyo, Japan) as described previously^53^.

### Oil Red O staining

The frozen liver samples with optimal cutting temperature were cryo-sectioned at 5 μm with a cryostat and then stained with Oil Red O working solution (Muto Pure Chemicals) for TG and FFA staining to evaluate hepatocyte steatosis^54^. Results were quantified using the WinRoof 7.4 software program (Mitani Corporation) as well.

### Immunohistochemical examinations

Immunohistochemical staining was performed on frozen sections using mouse anti-rat ED1 monoclonal antibody (Bio-Rad, Hercules, CA, USA) and Purified mouse anti-rat CD3 monoclonal antibody (BD Biosciences, San Diego, CA) as described previously^55^.

### Total mRNA preparation and quantitative reverse transcription polymerase chain reaction (qRT-PCR)

Total mRNA was extracted from liver tissues using an RNeasy Mini Kit (Qiagen, Valencia, CA, USA). Each 0.8-µg aliquot of mRNA was reverse-transcribed to cDNA using a Prime Script RT reagent Kit (RR037A; Takara, Shiga, Japan). qRT-PCR was performed by the SYBR® Green system using an Applied Biosystem PRISM7900 apparatus (Thermo Fisher Scientific, Waltham, MA, USA). The PCR cycle conditions for the SYBR^®^ Green system were 50 °C for 2 min, 95 °C for 2 min, 45 cycles of 95 °C for 15 s and 60 °C for 60 s. The comparative cycle threshold (CT) method was used to determine the relative gene expression. The results of target genes (Table 1) were normalized by subtracting the CT value of 18S expression. The fold change was calculated by a comparative CT method as described previously^56^.

**Table 1:** Primer sequences and probes used in this study.

Genes	Forward (5^**′**^–3^**′**^)	Reverse (5^**′**^–3^**′**^)	
SYBR green PCR system			
IFN-γ	GAAAGCCTAGAAAGTCTGAAGAAC	GCACCGACTCCTTTTCCGCTTCCT	
IL-6	TGATGGATGCTTCCAAACTG	GAGCATTGGAAGTTGGGGTA	
IL-1β	CACCTTCTTTTCCTTCATCTTTG	GTCGTTGCTTGTCTCTCCTTGTA	
CCR2	TTCTGGGCTCACTATGCTGC	AAGGGCCACAAGTATGCTGA	
Bax	CCAGGACGCATCCACCAAGAAGC	TGCCACACGGAAGAAGACCTCTCG	
Caspase-1	GTGTTGCAGATAATGAGGGC	AAGGTCCTGAGGGCAAAGAG	
Caspase-3	GGACCTGTGGACCTGAAAAA	GCATGCCATATCATCGTCAG	
Bcl-2	GGATGACTTCTCTCGTCGCTACCGT	ATCCCTGAAGAGTTCCTCCACCAC	
NFκB	GCATGCCATATCATCGTCAG	TGCTTCTCTCCCCAGGAATA	
OB-R	TGCCTTGGAGGACTATGGGT	AGCCCCCTTCAAAGACGAAG	
ACC	GCCTCTTCCTGACAAACGAG	TCCATACGCCTGAAACATGA	
SREBP-1c	TGGATTGCACATTTGAAGACAT	GCTCCTCTTTGATTCCAGGC	
FAS	CAGCTGTCAGTGTAAAGAAACATGTC	AGCTCACGTGCAGTTTAATTGTG	
HMGCR	CCCAGCCTACAAACTGGAAA	CCATTGGCACCTGGTACTCT	
SREBP-2	AGACTTGGTCATGGGGACAG	GGGGAGACATCAGAAGGACA	
18S	ATGAGTCCACTTTAAATCCTTTAACGA	CTTTAATATACGCTATTGGAGCTGGAA	

Genes	Forward (5^**′**^–3^**′**^)	Reverse (5^**′**^–3^**′**^)	Probe
Taqman probe PCR			
TNF-α	AATGGGCTCCCTCTCATCAGT	ACGGGCTTGTCACTCGAGTT	CCAGACCCTCACACTCAGATCATCTTCTCA
iNOS	GGACATTAACAACAACGTGGAGAA	AACCATTTTGATGCTTGTGACTCTT	TGCTATTCCCAGCCCAACAACACAGG
OPN	CAAAGTCCAGGAGTTTCCCTGTT	CTCTTCATGCGGGAGGTGA	TGATGAACAGTATCCCGATGCCACAGAT
18S	ATCCATTGGAGGGCAAGTCTGGTGC	ATGAGTCCACTTTAAATCCTTTAACGA	CTTTAATATACGCTATTGGAGGCTGGAA

### Western blot analyses

Western blot analysis was performed as described previously^20^. In brief, frozen liver tissues in the five groups were homogenized in RIPA buffer containing 1% protease inhibitor cocktail-1 and 1% protease inhibitor cocktail-2 (Sigma-Aldrich, St. Louis, MO, USA) followed by centrifugation in a microfuge at top speed for 30 min. Protein concentrations were assayed using a Bio-Rad Protein Assay (Bio-Rad). Samples were separated by electrophoresis on 10% polyacrylamide gels and transferred to Immobilon-PVDF (Bio-Rad). The membranes corresponding to the molecule of interest were cut out prior to hybridization with the antibody. After brief incubation with 5% non-fat milk to block non-specific binding, membranes were exposed overnight at 4 °C to specific caspase-1, caspase-3, caspase-9 and nuclear factor-κB (NF-κB). Protein expression was quantified by a laser densitometric analysis of the radiographic film using the ImageJ software program (NIH, Bethesda, MD, USA). The protein normalization was performed using β-actin as internal loading control.

### Statistical analyses

The GraphPad Prism 9 software program (GraphPad, San Diego, CA, USA) was used to calculate statistical significance. Student’s *t*-test was used for unpaired data. Data are expressed as the mean ± standard deviation (SD). A value of *p* < 0.05 was considered to be statistically significant (*p < 0.05; **p < 0.01; ***p < 0.001; ****p < 0.0001).

## Supplementary Information

Supplementary Figures.

## Data Availability

The datasets that support the findings of this study are available from the corresponding author on reasonable request.

## Abbreviations

ACC: Acetyl-CoA carboxylase
ALT: Alanine transaminase
AST: Aspartate transaminase
Bax: Bcl-2-associated X
CCR2: C–C chemokine receptor type 2
CDHCFF: Choline deficient high carbohydrate fat-free
FAS: Fatty acid synthase
FFAs: Free fatty acids
G2-SUISO: Coral calcium carried hydrogen G2
HCC: Hepatocellular carcinoma
H_2_: Hydrogen
HSCs: Hepatic stellate cells
IL-6: Interleukin 6
IL-1β: Interleukin-1 beta
IFN-γ: Interferon-gamma
iNOS: Inducible nitric oxide synthase
LDs: Lipid droplets
mRNA: Messenger RNA
NASH: Non-alcoholic steatohepatitis
NAFLD: Non-alcoholic fatty liver disease
NFκB: Nuclear factor-κB
OB-R: Leptin receptor
OPN: Osteopontin
ROS: Reactive oxygen species
RT-PCR: Real time-polymerase chain reaction
SREBP-1c: Sterol regulatory element-binding protein-1c
TC: Total cholesterol
TNF-α: Tumor necrosis factor-alpha
