# Supplementary material for: Coral calcium carried hydrogen ameliorates the severity of non-alcoholic steatohepatitis induced by a choline deficient high carbohydrate fat-free diet in elderly rats
Source: Sci Rep. 2023 Jul 19;13:11646. doi: 10.1038/s41598-023-38856-6 (PMC10356788; doi:10.1038/s41598-023-38856-6)
Supplement: Supplementary file 1 — Supplementary Figures. [file 41598_2023_38856_MOESM1_ESM.pptx]

## Slide 1
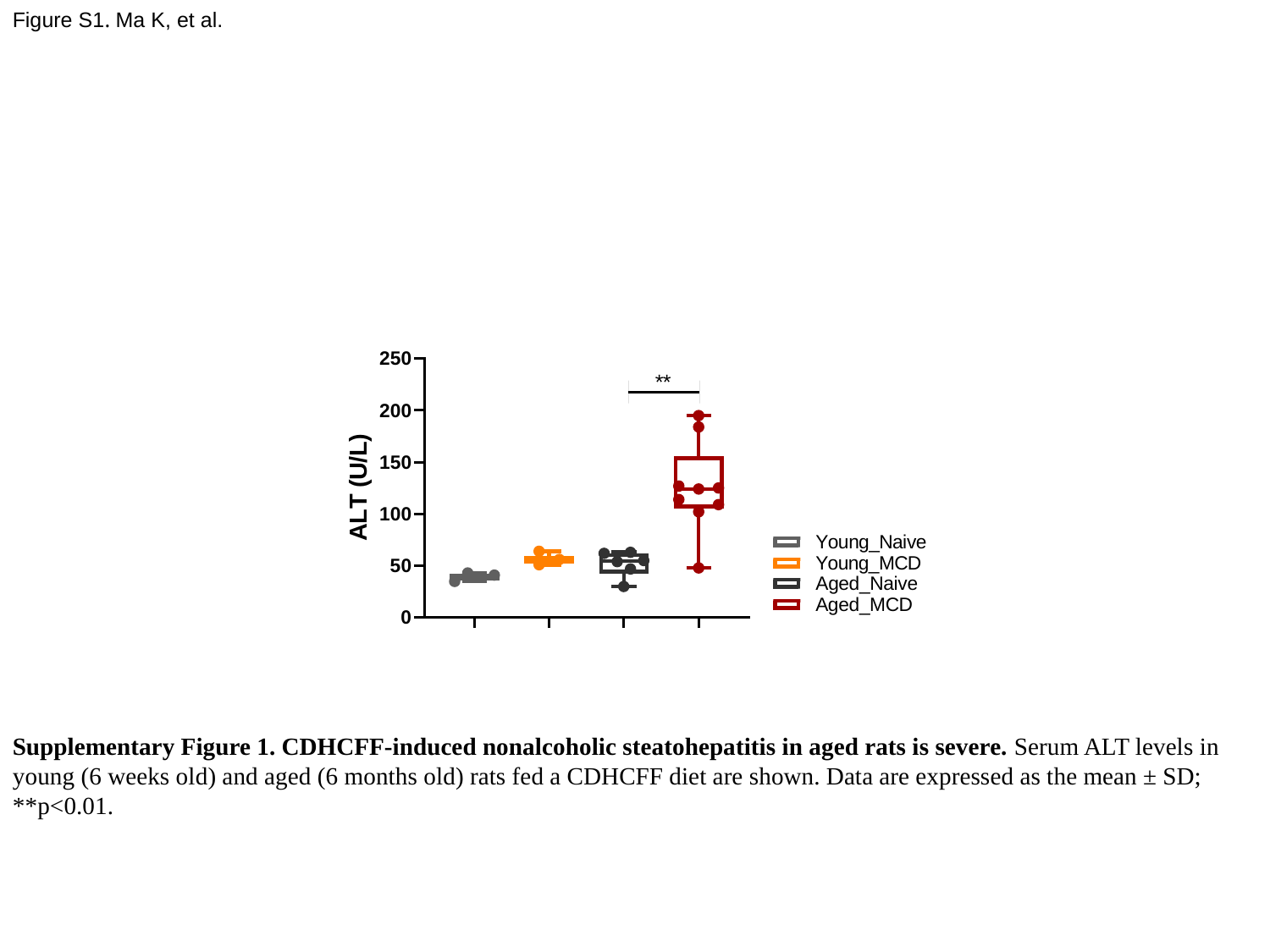

Figure S1. Ma K, et al.
Supplementary Figure 1. CDHCFF-induced nonalcoholic steatohepatitis in aged rats is severe. Serum ALT levels in young (6 weeks old) and aged (6 months old) rats fed a CDHCFF diet are shown. Data are expressed as the mean ± SD; **p<0.01.

## Slide 2
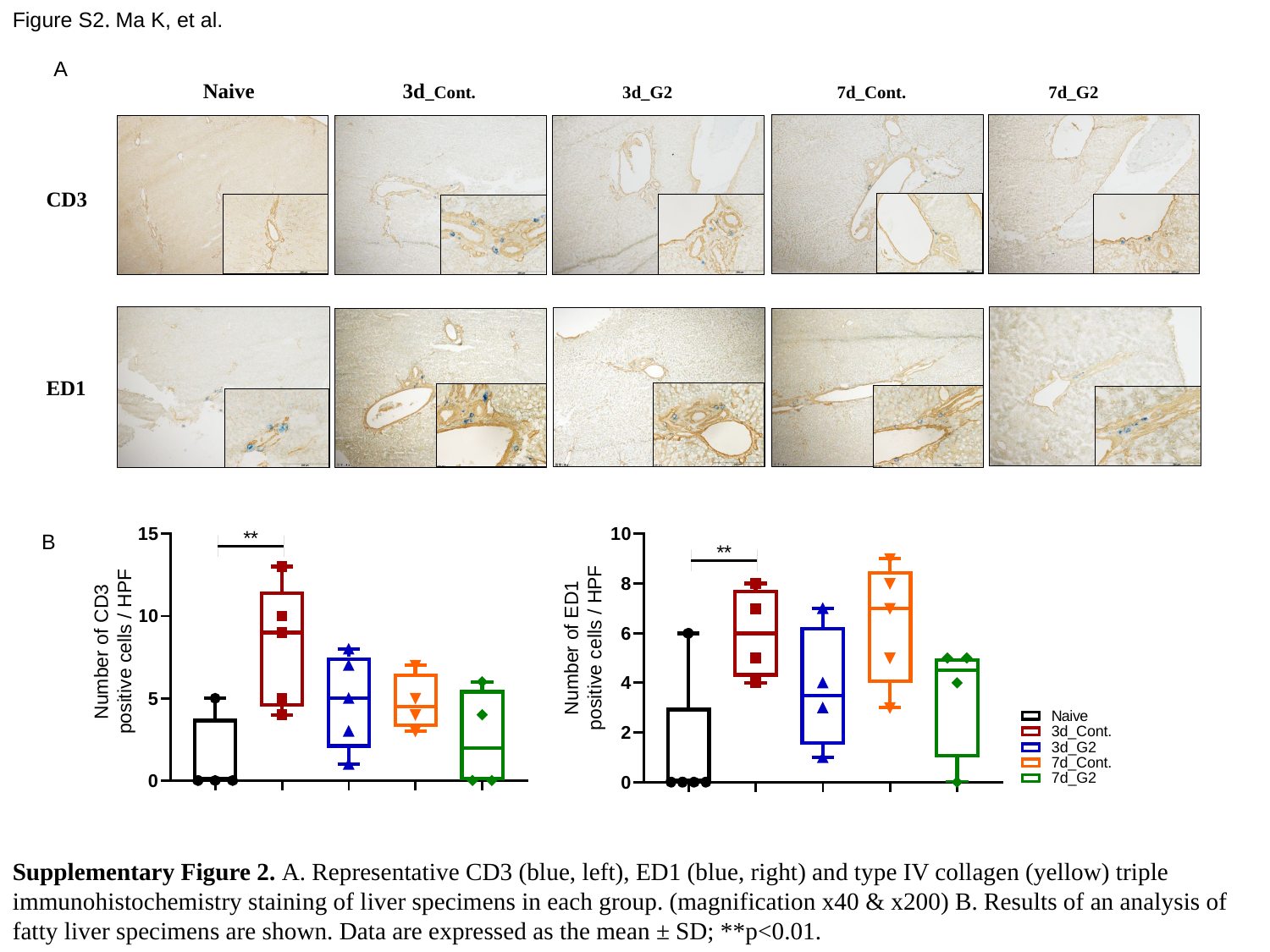

Figure S2. Ma K, et al.
A
 Naive 3d_Cont. 3d_G2 7d_Cont. 7d_G2
CD3
ED1
B
Number of ED1 positive cells / HPF
Number of CD3 positive cells / HPF
Supplementary Figure 2. A. Representative CD3 (blue, left), ED1 (blue, right) and type IV collagen (yellow) triple immunohistochemistry staining of liver specimens in each group. (magnification x40 & x200) B. Results of an analysis of fatty liver specimens are shown. Data are expressed as the mean ± SD; **p<0.01.

## Slide 3
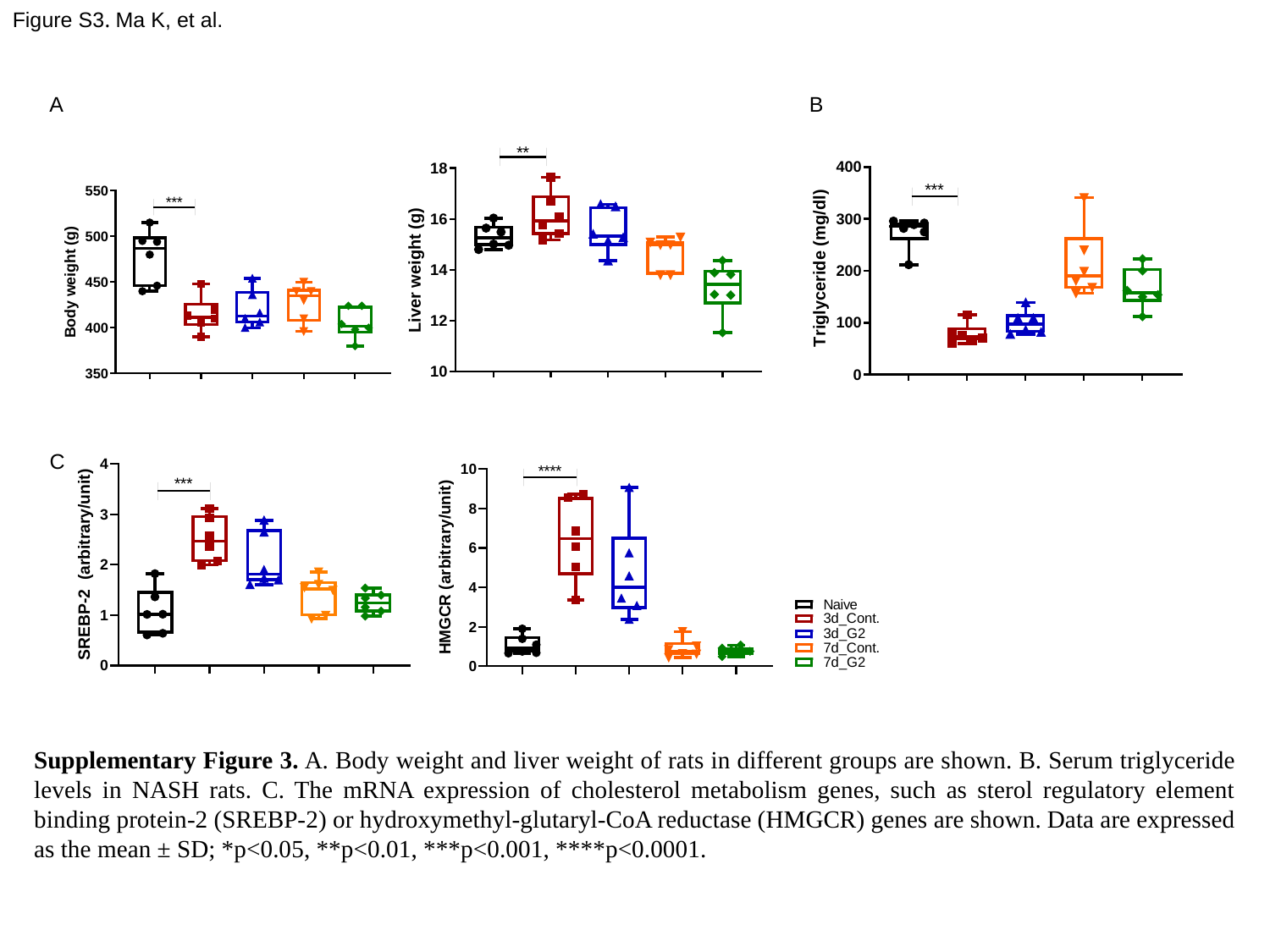

Figure S3. Ma K, et al.
A
B
C
Supplementary Figure 3. A. Body weight and liver weight of rats in different groups are shown. B. Serum triglyceride levels in NASH rats. C. The mRNA expression of cholesterol metabolism genes, such as sterol regulatory element binding protein-2 (SREBP-2) or hydroxymethyl-glutaryl-CoA reductase (HMGCR) genes are shown. Data are expressed as the mean ± SD; *p<0.05, **p<0.01, ***p<0.001, ****p<0.0001.

## Slide 4
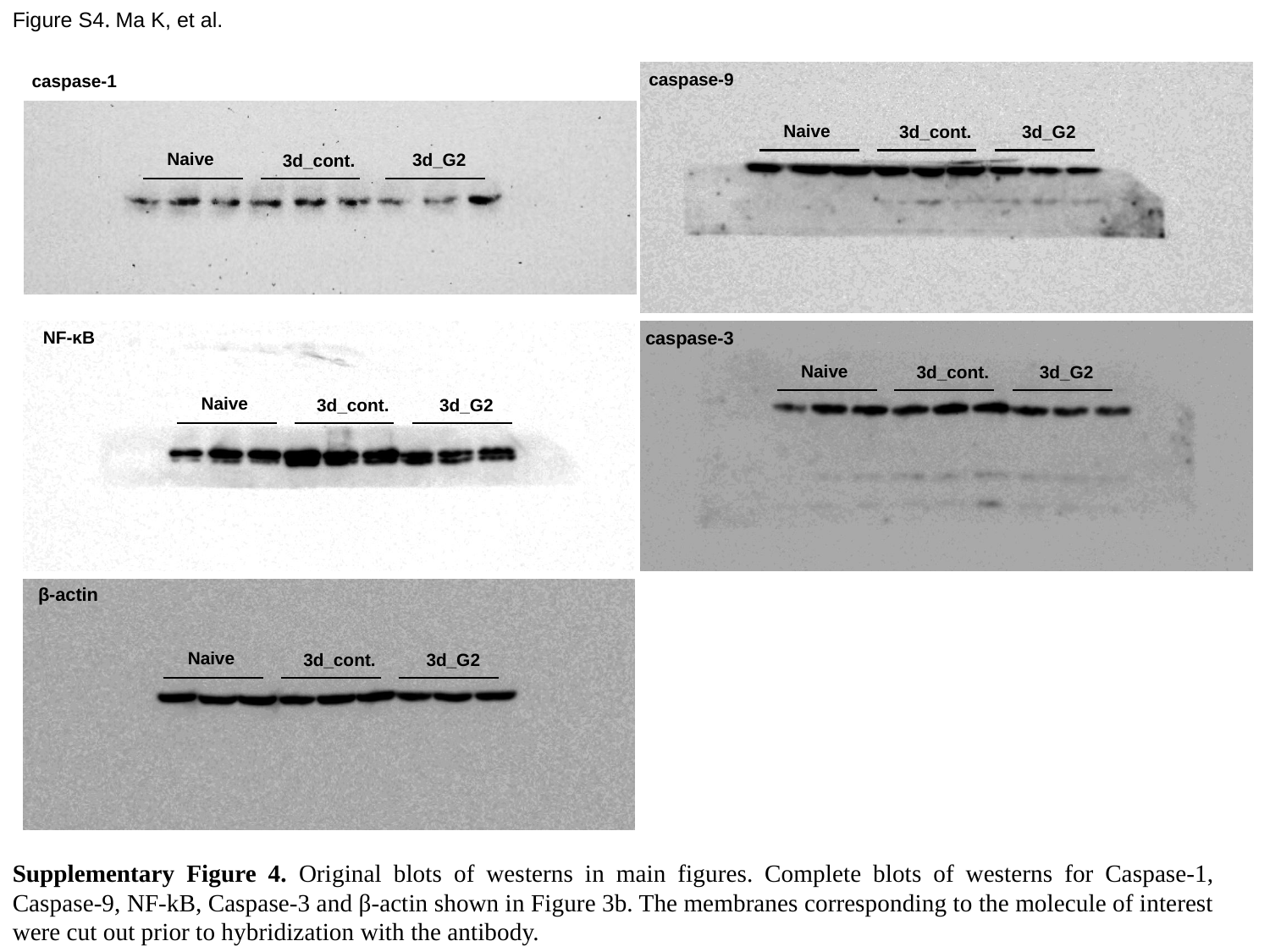

Figure S4. Ma K, et al.
caspase-9
caspase-1
Naive
3d_cont.
3d_G2
Naive
3d_cont.
3d_G2
NF-κB
caspase-3
Naive
3d_cont.
3d_G2
Naive
3d_cont.
3d_G2
β-actin
Naive
3d_cont.
3d_G2
Supplementary Figure 4. Original blots of westerns in main figures. Complete blots of westerns for Caspase-1, Caspase-9, NF-kB, Caspase-3 and β-actin shown in Figure 3b. The membranes corresponding to the molecule of interest were cut out prior to hybridization with the antibody.
